# Visualization of Light Propagation with Multifocal Intraocular Lenses Using the Ouzo Effect

**DOI:** 10.1155/2019/6425040

**Published:** 2019-06-27

**Authors:** Timo Eppig, Kathrin Rubly, Antonia Rawer, Achim Langenbucher

**Affiliations:** ^1^Institute of Experimental Ophthalmology, Saarland University, 66421 Homburg, Saar, Germany; ^2^AMIPLANT GmbH, Haidling 1, 91220 Schnaittach, Germany; ^3^School of Engineering, Hochschule für Technik und Wirtschaft des Saarlandes, University of Applied Sciences, Goebenstr. 40, 66117 Saarbrücken, Germany; ^4^School of Mechanical Engineering and Process Engineering, Clausthal University of Technology, Leibnizstr. 2, 38678 Clausthal-Zellerfeld, Germany

## Abstract

The number of presbyopia correcting intraocular lenses (IOLs) is increasing and new technologies are constantly emerging with the aim of correcting the loss of accommodation after cataract surgery. Various optical designs have been proposed to implement multifocality or an extended depth of focus (EDOF). Depending on the optical principle of an implanted lens, the visual performance often is deteriorated by superposition of individual image planes and halos of varying intensity. This experimental study presents a concept to visualize the light fields and especially the halos of mono- and multifocal IOLs using the well known alcoholic beverage “ouzo” in order to obtain qualitative data on the imaging characteristics. We conclude that ouzo is a useful, cost effective, and nonpolluting medium for beam visualization and an alternative to fluorescein or milk, which could find an application for educational purposes.

## 1. Introduction

Apart from monofocal intraocular lenses (IOLs) that generate a single focus in a specific distance, there are different ways to generate two or more foci by various optical principles. Multifocal lenses statically provide two or more foci at distinct distances at the same time in order to provide spectacle independence to the patient for distance and near vision [1]. Combinations of the diffractive optics in terms of Fresnel zone plates and refractive properties of the optical material represent the most common type of multifocal IOL. Purely refractive multifocal lenses have also been presented; examples hereof are the ReZoom™ IOL (American Medical Optics, Santa Ana, USA) and the more recently presented Lentis® MPlus (Oculentis GmbH, Berlin, Germany) or the segmented bifocal lenses SBL-2 and SBL-3 (Lenstec, Inc., St. Petersburg, FL, USA). The design of the ReZoom™ IOL was based on concentric annular zones with alternating refractive power, whereas the Lentis® MPlus has a nonrotationally symmetric segmented design [2]. A quite new concept is implemented in the Tecnis® Symfony® IOL (Johnson & Johnson Vision, Santa Ana, USA), which is basically a diffractive multifocal IOL which aims to provide an extended depth of focus (EDOF) [3–5]. Other concepts such as refractive EDOF lenses [6], the light-sword lens [7], small aperture implants [8], and accommodating IOLs have also been proposed [9]. A specific amount of light is “lost” to (unused) higher diffractive orders when using Fresnel zone plates. These higher diffractive orders do not contribute to the image formation, but the light reaches the retinal plane. The superposition of the individual images and the unused light from higher diffractive orders cause formation of halos and a degradation of image contrast (sometimes referred to as “waxy vision”) [10–12]. These halos are often reported by patients [10, 13] but still, many patients are satisfied with the visual performance of multifocal IOLs. It is well known that visual performance with multifocal lenses improves within the first months after surgery due to neural adaptation to the altered visual sensation [14, 15]. Kaymak et al. showed that training might accelerate this adaptation phase [15]. Some patients, however, suffer from persistent visual disturbance limiting their quality of life. In some cases, multifocal IOLs have to be explanted due to persistent visual discomfort and substituted with a monofocal IOL [16, 17].

Several researchers provided images showing the light propagation of multifocal lenses in order to improve comprehension of the image formation and inevitable image superposition. These authors mostly used milk powder [18] or fluorescein [19, 20] as scattering/fluorescence medium to visualize the light emerging from the IOL. Ouzo is a famous traditional alcoholic aniseed-flavored beverage originating from Greece. Similar alcoholic beverages are common around the Mediterranean sea, such as “Pastis” in France, “Sambucca” in Italy, or “Raki” in Turkey. Ouzo is well known to create the so-called “ouzo effect” [21] when dissolved in water: although both water and ouzo are clear liquids, the mixture of both looks milky. This effect is caused by dispersion of microdroplets of oil in a solvent; the size of the droplets is typically something between 0.3 *μ*m and 1.5 *μ*m in diameter [22]. Such emulsions may be stable for a long time period and are used in a variety of technical applications [23]. We therefore hypothesized that an ouzo-water blend may be a useful medium for light visualization.

The purpose of this study was to implement an experimental procedure in order to characterize the halos of mono- and multifocal IOLs and in order to obtain qualitative information on the image characteristics. This work describes the development of such a setup and presents first results along with an interpretation of the results.

## 2. Methods

**Figure 1:**
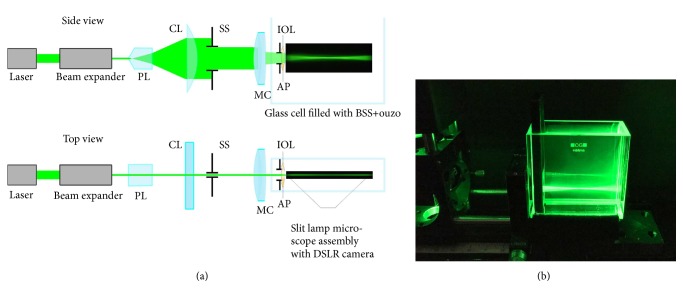
(a) Sketch of the experimental setup. A Powell lens is used to expand the laser beam which is collimated in one dimension by a cylindrical lens (CL). A slit stop (SS) is placed behind the cylinder lens to form a rectangular beam shape. The collimated fan is passing the model cornea and the intraocular lens (IOL). (b) The light that emerges in the IOL is scattered in the medium containing ouzo making the light path visible.

**Figure 2:**
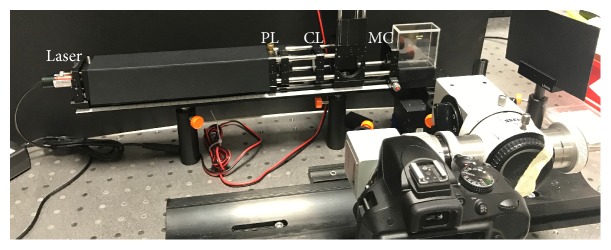
Photograph of the experimental setup showing the optical pathway with the laser, Powell lens (PL), cylinder lens (CL), and model cornea (MC). The assembly in the front shows the camera attached to a slit lamp microscope assembly.

The methods were adopted from Reiss et al. [19]. The setup comprises a monochromatic line light source, an eye model, and an image acquisition system. The image acquisition system includes a consumer grade digital single-lens reflex (DSLR) camera (D3300, Nikon Corp., Tokio, Japan) and the microscope unit of an ophthalmological slit lamp (SL30, Carl Zeiss Meditec AG, Oberkochen, Germany) (Figure 1). A diode-pumped solid state laser module with a wavelength of 532 nm (CW532-30, Roithner Lasertechnik GmbH, Austria) and a beam diameter of 1.5 mm is being used as light source. A reversed beam expander further reduces the laser beam diameter and a Powell lens (laser line generator #43-473, Edmund Optics GmbH, Karlsruhe, Germany) generates a divergent laser line with homogenous intensity distribution. A cylindrical lens (CL, f=40 mm) then collimates the laser fan in one dimension (Figure 1(a)). A slit stop (SS, 0.3 mm width) is used to form a rectangular laser line. The eye model's components are an achromatic doublet (LAO0434, Melles Griot BV, Didam, The Netherlands) serving as model cornea, according to ISO 11979-2:2014 [24] and the IOL under test in a cuvette (700-000-20-10, Hellma GmbH & Co. KG, Müllheim, Germany). The cuvette is filled with balanced saline solution (BSS, Ringer's solution, Baxter Deutschland GmbH, Unterschleißheim, Germany) and an aniseed-flavoured alcoholic drink (Ouzo 12, 38 vol.-% alcohol, Kaloyiannis-Koutsikos Distillers S.A., Volos, Greece). An aperture stop (AP, *Ø*=4.5 mm) is placed directly in front of the IOL in order to simulate a physiological pupil. Positioning of the sample within the cuvette is managed with a special IOL holder (Rotlex (1994) Ltd., Omer, Israel), and the cuvette itself is placed on a custom stage 3D-printed from polyactide (PLA) by a consumer grade 3D printer (Ultimaker 2Go, Ultimaker B.V., Geldermalsen, The Netherlands). The custom stage with the cuvette was placed on a linear stage allowing proper centration of the IOL relative to the beam. A photograph of the experimental setup is shown in Figure 2.

### 2.1. Image Acquisition and Analysis

Images were taken with the DSLR camera via USB using external software (digiCamControl [25]) to minimize vibration to the image acquisition apparatus during exposure. We used a microscope magnification of 12× for taking the images with the IOLs. The acquired raw photographs were loaded into MATLAB (The MathWorks, Inc., Natick, USA) and vertically stretched by a factor of four. Then we analyzed the axial light distribution at the brightest row in the image and determined the locations of the foci. We used Gaussian smoothening in order to reduce noise in the image. The axial and the lateral light distribution in the foci were plotted to determine the magnitude of light surrounding the foci in order to allow an estimate for the halo.

### 2.2. Visualization Medium

**Figure 3:**
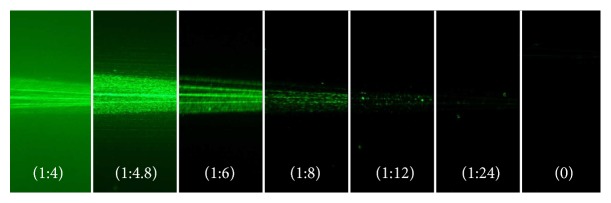
Illustration of the image contrast with various ouzo-water blends and a multifocal intraocular lens (intraocular lens is placed on the left side). The image brightness increases with the amount of ouzo until the number of aniseed oil droplets becomes too large causing the image contrast to decrease.

Before taking images with the IOLs, we determined the optimal ouzo concentration in pure water for best image contrast (Figure 3). Therefore, we placed an IOL into a glass cell. The initial amount of water was 240 ml and we subsequently added 10 ml of ouzo to the cuvette while observing the image contrast and quality.

### 2.3. Intraocular Lenses

Five IOLs with different optical concepts were analyzed: one monofocal aspheric lens, a diffractive and a asymmetric segmented refractive bifocal IOL, a diffractive EDOF lens, and a diffractive trifocal IOL with EDOF (Table 1).

## 3. Results

**Figure 4:**
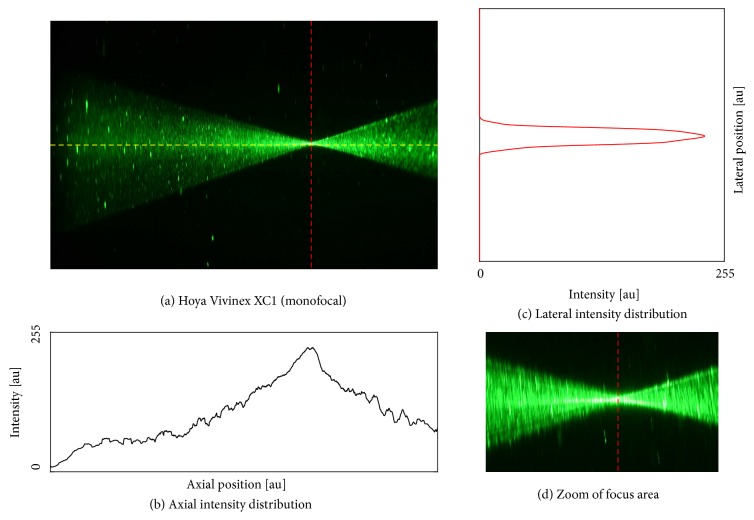
Hoya Vivinex® XY1 (monofocal). (a) Raw image along with the axial intensity profile (b) and the lateral intensity distribution at the best focus (c). Subfigure (d) shows the magnified focus area.

**Figure 5:**
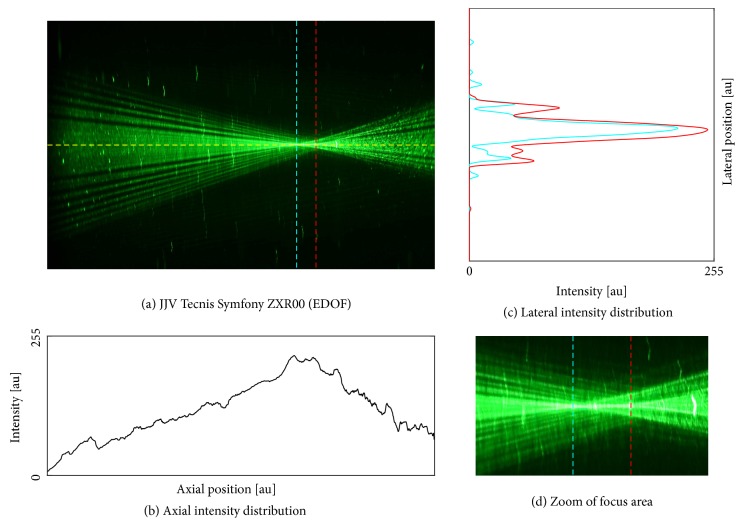
Johnson & Johnson Vision Tecnis® Symfony® ZXR00 (EDOF). (a) Raw image along with the axial intensity profile (b) and the lateral intensity distribution at the best focus (c). Subfigure (d) shows the magnified focus area.

**Figure 6:**
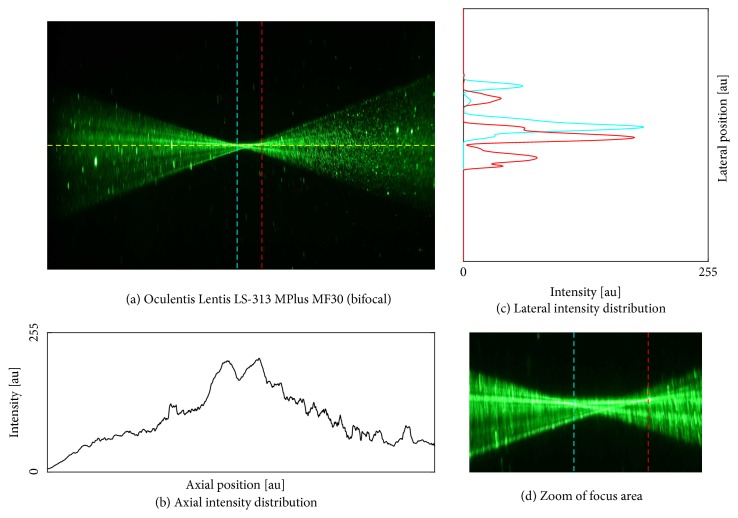
Oculentis Lentis® MPlus MF30 (refractive bifocal). (a) Raw image along with the axial intensity profile (b) and the lateral intensity distribution at the best focus (c). Subfigure (d) shows the magnified focus area.

**Figure 7:**
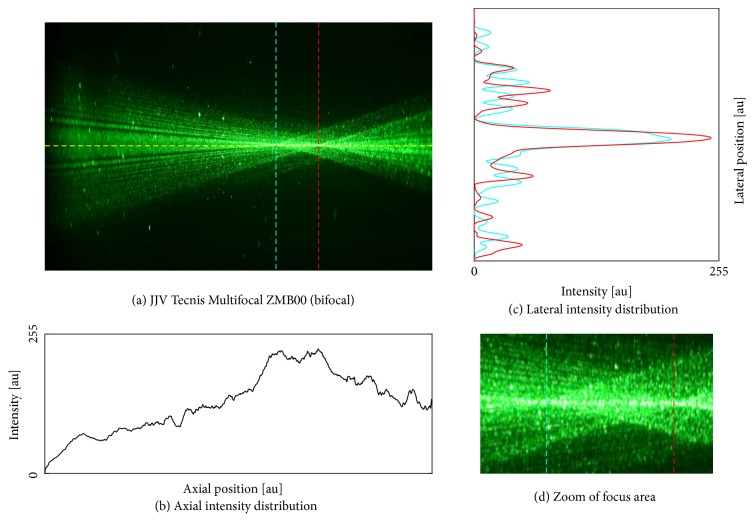
Johnson & Johnson Vision Tecnis® Multifocal ZMB00 (bifocal). (a) Raw image along with the axial intensity profile (b) and the lateral intensity distribution at the best focus (c). Subfigure (d) shows the magnified focus area.

**Figure 8:**
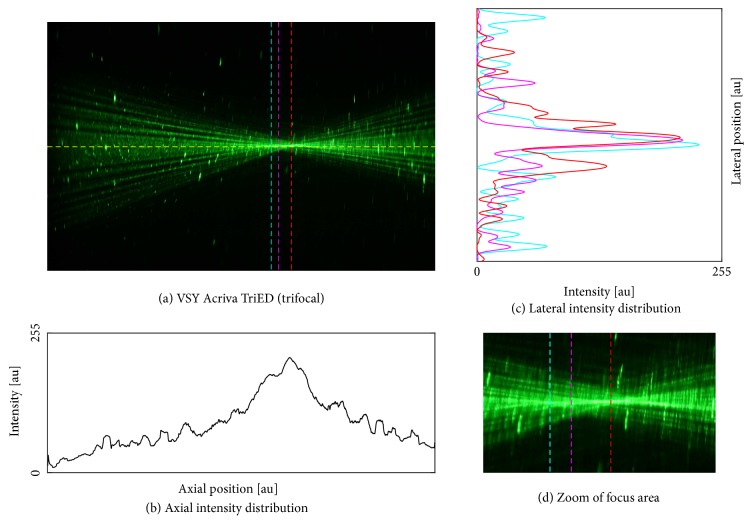
VSY Acriva® Tri-ED (trifocal). (a) Raw image along with the axial intensity profile (b) and the lateral intensity distribution at the best focus (c). Subfigure (d) shows the magnified focus area revealing some motion artifacts originating from floating oil droplets.

We found an optimal image contrast with a concentration of 10.7% ouzo (3 ml mixed with 25 ml BSS). We proceeded with the IOLs using this ouzo concentration. Photographs of the five different samples are shown in Figures 4–8. The monofocal IOL shows a single distinct focus (Figure 4) without any surrounding halos, whereas the EDOF IOL did not show a distinct sharp focus (Figure 5). The multifocal lenses exhibited the expected number of focal points. The refractive bifocal IOL (Figure 6) showed asymmetric light cones with a superior located near distance focus and an inferior located far distance focus (note that this is arbitrary, as we did not take care of proper up/down placement). Thus both images will not be concentric but overlapping in a decentered way. Clinical results of this IOL indicate that the placement of the near addition zone does not affect the visual outcome [26]. The diffractive bifocal lens showed two distinct coaxial foci (Figure 7). Halos could be “seen” around the individual focal points in all multifocal lenses including the EDOF lens. Halos seemed to be more prominent in the trifocal lens (Figure 8) than in the bifocal lens (Figure 7) and in the EDOF lens (Figure 5). The diffractive lenses showed symmetric halos around the foci (Figures 5, 7, and 8), whereas the halo of the refractive bifocal lens was asymmetric (Figure 6).

## 4. Discussion

With this setup we were able to visualize different concepts of multifocal IOL, showing the working principle of a nonrotationally symmetric refractive multifocal IOL in comparison to the more commonly used diffractive multifocal IOL principle. The monofocal and bifocal IOLs revealed the expected amount of focal points: the monofocal IOL shows a single sharp focus without any surrounding halos. With the bifocal and the EDOF IOLs, two foci could be identified which were both surrounded by defocused light from the complementary focus. With the trifocal lens, the three foci could not be clearly identified from the axial distribution and the halos seemed to be more prominent than in the bifocal and EDOF lenses. A direct comparison of the amount of halos, however, is not possible as the site and intensity of the halos are depending on pupil diameter, base power, and addition power of the IOL [27]. This is also a major limitation of the current work, as the lenses under test had different base powers (and addition powers). The pupil diameter, however, was fixed. Further experiments with IOL of similar base power should provide better information on the extent of the halos between the lenses.

**Figure 9:**
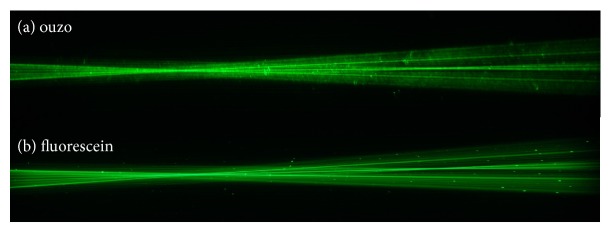
A comparison of a multifocal IOL immersed in two different visualization media: ouzo in water (a) vs. fluorescein in water (b) both illuminated with a green laser (*λ*=532 nm). The medium containing ouzo (a) shows more Schlieren in the image, while the medium containing fluorescein (b) seems to provide a sharper image. Scattering from particles in the medium is visible in both images (adopted from [28]).

**Figure 10:**
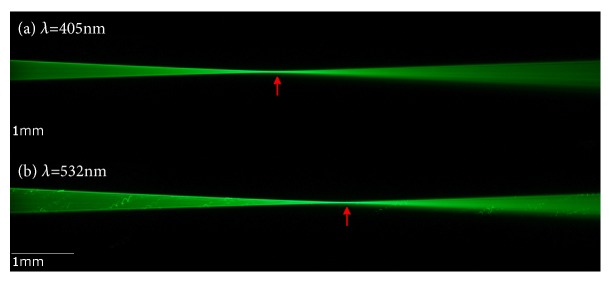
A comparison of a monofocal lens immersed in a medium containing fluorescein and illuminated with two different wavelengths 405 nm (a) and 532 nm (b). Both images show a green signal because of the green flourescent reaction of the fluorescein. Image (a) is based purely on fluorescence (excitation with 405 yields green emission (see also Figure 11). Image (b) is based on fluorescence and scattering exposing particles in the medium (mixed excitation and emission light). The red arrows indicate the estimated location of the focus: the IOL shows a shorter focal length (higher refractive power) with the short wavelength (a) compared to the medium wavelength (b) (adopted from [28]).

**Figure 11:**
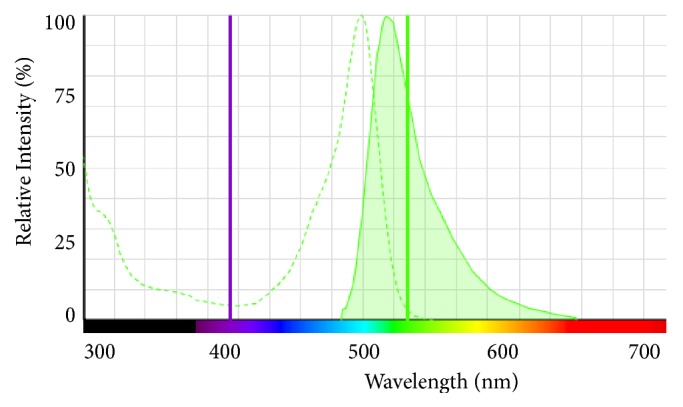
Simulated excitation (dashed) vs. emission (solid) spectra of fluorescein (FITC) along with two laser lines at 405 nm and 532 nm (image created with ThermoFisher SpectraViewer [29]).

The use of ouzo as a visualization medium for the light path created by various IOLs is a straightforward concept which could be used in any educational experiment. Sitnikova et al. found that an ouzo-water-emulsion may stay stable for several months [23] and does not suffer from photodegradation, which makes it a useful test medium. Other dilutions like milk powder [18] or fluorescein [19, 20], which have been used in previous publications, may degrade or segregate from water over time. However, the image quality was impaired by Schlieren and frequent bright spots/stripes originating from saline crystals (as they were seen in pure BSS and fluorescein in BSS as well, compare Figure 9), dust, or oil droplets. The stripes originate from the relatively long exposure time (1/4 s) for taking the photographs. Due to the low concentration of ouzo, multiple scattering or absorption did perturb the measurements. The slit stop caused some amount of diffraction, but due to the low intensity of the additional maxima no effect on the photograph quality could be observed. Since scattering media like milk or ouzo are independent of the wavelength used in the setup, the analysis could be performed with virtually any wavelength of light. Therefore, it might also be useful for investigating dispersive properties of intraocular lenses. Other visualization media, e.g., fluorescent dyes such as fluorescein, have the advantage of less Schlieren and scattering effects when they are used in fluorescent mode only (compare Figures 9 and 10) but they are highly dependent on the wavelength of the excitation light. Reiss et al. and Son et al. [19, 20] used fluorescein in combination with a green laser which does not address the full quantum efficiency of the fluorescein (Figure 11). Therefore higher laser intensity is required which also makes scattered light visible. The optimum excitation wavelength would be approx. 515 nm which uses the full quantum efficiency of the fluorescein requiring less laser intensity. We used laser with 405 nm instead, which addresses a higher quantum efficiency with the fluorescein than with 532 nm and excitation/emission light could be optically separated by optical filters. However, 405 nm is a less interesting wavelength in terms of visual perception as the retina's sensitivity is about ten times less than that of green light. We also experimented with fluorescein using the two wavelengths 532 nm and 405 nm which allowed us to visualize the dispersion of an intraocular lens by switching between both light sources with the IOL remaining in place (Figure 10). These experiments were performed without model cornea and with a larger cuvette in order to stretch the light path.

Another limitation of this work is that these images do not reflect the reality in the human eye, where all focal points will be superimposed because of different object distances. These images can just provide an insight in the underlying optic principle of various IOLs. In addition, the image quality was insufficient for any quantitative investigation on the light distribution. Therefore, our method is not suitable for image quality assessment, and it could only provide an estimation on the expected amount of halos and does not correlate with the actual halos that might be perceived by a patient. In a following study, we developed a modified setup and method [30], which will allow a distinct separation of light contributing to the individual focal points for near and far distance vision.

There have been other methods and test devices proposed that allow for a detailed analysis of the imaging quality of monofocal and multifocal IOLs. These methods are mostly based on imaging of a point light source [31–34] onto a camera. An attached computer system is then used to derive the modulation transfer function (MTF) from the point spread function (PSF) in order to quantify the imaging properties of an IOL. These methods are based on basic optical systems theory and have been implemented in several commercially available devices, such as the OPAL Vector System (Image Science Ltd., Oxford, United Kingdom), the PMTF (Lambda-X S.A., Nivelles, Belgium), and the OptiSpheric IOL (TRIOPTICS GmbH, Wedel, Germany). Although these methods are very precise in quantifying the image quality of IOLs, they can only provide limited information on the formation of halos or the light propagation by recording through-focus PSF/MTF data. Other methods use extended objects such as slit/cross targets or bar/letter charts to be imaged through an IOL [35–38]. These charts allow for a better comprehension of the visual effects on image quality including the effects of halos on the image quality. The measurability of image quality with bar or letter charts is limited but comparability to visual acuity results may be better. Even more intuitive but with limited measurability are systems used to “simulate” patients' vision after implantation of an IOL; such systems have been proposed by Eisenmann et al. [39], Kusel & Rassow [40], and Pujol et al., which was implemented in the VirtIOL device [41, 42]. These methods allow for a psychophysical estimation of image quality and extent of halos and are especially interesting for patient consultation prior to (multifocal) IOL implantation.

As a conclusion we find that ouzo is a useful, cost effective, and nonpolluting medium for beam visualization and an alternative to fluorescein or milk. However, the macroscopic oil droplets lead to inhomogeneous illumination of the beam which limits the usability for quantitative measures. Therefore, the ouzo method may primarily be used for educational purposes to help understand the principles of multifocal intraocular lenses. Other applications include educational projects for visualizing beam propagation in addition to the analysis of image quality.

## Figures and Tables

**Table 1:** List of intraocular lenses (IOLs) analyzed in this study (EDOF: extended depth of focus).

Manufacturer	Intraocular lens type	Power [D]	Optical principle
Hoya Surgical Optics GmbH	Vivinex® XY1	20.5	refractive monofocal
Frankfurt, Germany			

Johnson & Johnson Vision	Tecnis® Multifocal ZMB00	20.0 +4.0	diffractive bifocal
Santa Ana, CA, USA	Tecnis® Symfony® ZXR00	20.0 +1.75	diffractive EDOF

Oculentis GmbH	Lentis® Mplus LS-313 MF30	24.5 +3.0	asymmetric segmented
Berlin, Germany			refractive bifocal

VSY Biotechnology BV	Acriva^UD^ Reviol Tri-ED	n/a +3.0/+1.5	diffractive trifocal
Amsterdam, The Netherlands			

## Data Availability

The data used to support the findings of this study are included within the article.
